# CNN3 acts as a potential oncogene in cervical cancer by affecting RPLP1 mRNA expression

**DOI:** 10.1038/s41598-020-58947-y

**Published:** 2020-02-12

**Authors:** Lili Xia, Yongfang Yue, Mingyue Li, Ya-Nan Zhang, Lu Zhao, Weiguo Lu, Xinyu Wang, Xing Xie

**Affiliations:** 1 Women’s Reproductive Health Laboratory of Zhejiang Province, Women’s Hospital, School of Medicine, Zhejiang University, Hangzhou, 310006 Zhejiang China; 2 Department of Gynecologic Oncology, Women’s Hospital, School of Medicine, Zhejiang University, Hangzhou, 310006 Zhejiang China

**Keywords:** Cancer, Cell invasion, Metastasis

## Abstract

The prognosis of advanced stage cervical cancer is poorer due to cancer invasion and metastasis. Exploring new factors and signalling pathways associated with invasiveness and metastasis would help to identify new therapeutic targets for advanced cervical cancer. We searched the cancer microarray database, Oncomine, and found elevated calponin 3 (CNN3) mRNA expression in cervical cancer tissues. QRT-PCR verified the increased CNN3 expression in cervical cancer compared to para-cancer tissues. Proliferation, migration and invasion assays showed that overexpressed CNN3 promoted the viability and motility of cervical cancer cells, the opposite was observed in CNN3-knockdown cells. In addition, xenografted tumours, established from SiHa cells with CNN3 knockdown, displayed decreased growth and metastasis *in vivo*. Furthermore, RNA-sequencing showed that ribosomal protein lateral stalk subunit P1 (RPLP1) was a potential downstream gene. Gene function experiments revealed that RPLP1 had the same biological effects as CNN3 did. Rescue experiments demonstrated that the phenotypes inhibited by CNN3 silencing were partly or completely reversed by RPLP1 overexpression. In conclusion, we verified that CNN3 acts as an oncogene to promote the viability and motility of cervical cancer cells *in vitro* and accelerate the growth and metastasis of xenografted tumours *in vivo*, by affecting RPLP1 expression.

## Introduction

Cervical cancer is the fourth most common malignancy and the fourth leading cause of cancer death in women worldwide, with an estimated 570,000 new cases and 311,000 deaths globally in 2018^1^. Due to unpopularization of screening in developing countries many patients are diagnosed at the advanced disease stage, with metastases of lymph nodes and other organs at their initial visit. Unfortunately, more than 20% of these cases relapse because of unsuitability as candidates for surgery or insensitivity to radiotherapy or chemotherapy^2–4^, and the 5-year survival rate of cervical cancer patients with distant metastasis is approximately 17%^5^.

Invasiveness and metastasis are the main features and cause of death in cancers with advanced stages. Multiple processes, factors, and signalling pathways have been identified to be associated with cancer invasiveness and metastasis, such as the epithelial-mesenchymal transition, cancer stem cells, circRNAs, and the Hippo-YAP and MAPK pathways, but few of them have been translated into effective treatment strategies in the clinic^6,7^. Finding new targets to block cancer invasion and metastasis would help improve the prognosis of advanced cervical cancer patients. Therefore, we searched the Oncomine database and observed an elevated mRNA expression of CNN3 in cervical cancer versus normal cervix tissues^8^. Calponin 3, hereafter abbreviated as CNN3, is one of the members of the evolutionarily highly conserved actin-binding protein family and is involved in cytoskeletal rearrangement. CNN3 was initially found to function in regulating neural tube morphogenesis in embryo development^9^. Systematic knockout of CNN3 in mice caused embryonic and neonatal death due to defects in the development of the central nervous system^10^. Subsequent research provided evidence for CNN3 functions beyond neurogenesis, such as the cellular fusion of trophoblasts and myoblasts, the cell motility and contractile ability of dermal fibroblasts, and others, in which CNN3 plays a role in cell differentiation, proliferation and migration via stress fibre formation or cytoskeletal remodelling^11–13^. Recently, studies also showed that CNN3 was related to lymph node and peritoneal metastasis in colorectal cancer^14,15^. However, the effect of CNN3 on metastasis in malignancies other than colorectal cancer is unknown to date.

In the present study, we verified the CNN3 overexpression in cervical cancer tissues, and its role in promoting proliferation, migration, and invasion in cervical cancer cells and accelerating the growth and metastasis of xenografted tumours in immunodeficient mice. Further, we identified ribosomal protein lateral stalk subunit P1, also known as RPLP1, is controlled transcriptionally by CNN3, and participates in the CNN3-mediated regulation of the viability and motility of cervical cancer cells. Our findings provide evidence that CNN3 acts as an oncogene and may serve as a potential target for blocking the metastasis of cervical cancer.

## Results

### Highly expressed CNN3 promotes the viability and motility of cervical cancer cells

**Figure 1:**
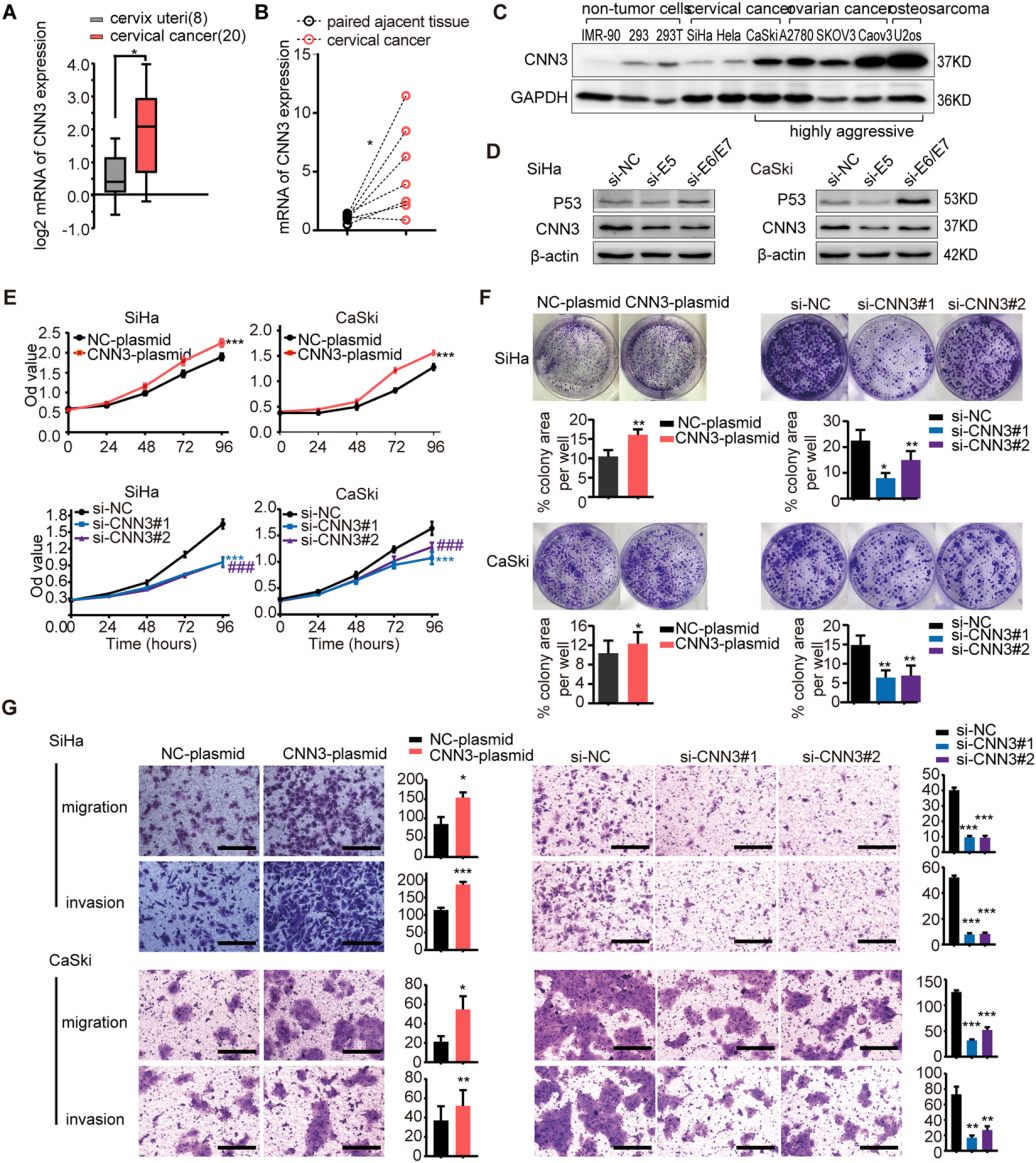
Highly expressed CNN3 promotes the viability and motility of cervical cancer cells. (**A**) Box plots of CNN3 mRNA expression in 20 cervical cancer tissues and 8 normal cervix tissues from Oncomine database of Pyeon Multi-cancer (*P < 0.05). (**B**) The mRNA expression of CNN3 in 7 pairs of cervical cancer and adjacent tissues by RT-PCR (*P < 0.05). Paired adjacent non-cancerous tissues were obtained at 3 cm away from the tumour edge and all tissues were reviewed by an experienced pathologist (Dr. Xiaofei Zhang). (**C**) CNN3 protein expression were then determined in 10 cell lines with immunoblot analysis. The grouping of blots cropped from same parts of the same gel. Full-length blots are presented in Supplementary Fig. S3. (**D**) SiHa and CaSki cells were transfected with si-NC, si-E5 and si-E6/E7 siRNA for 48 h. The expressions of P53 and CNN3 protein were detected by immunoblot analysis. The expressions of P53, a downstream molecule of 16E6, were tested to confirm the interference efficiency of si-E6/E7^27,28^. Blots of CNN3 and β-actin cropped from same parts of the same gel, while blots of P53 cropped from different parts of gel. Full-length blots are presented in Supplementary Fig. S3. (**E**) Cell viability of SiHa and CaSki was detected by CCK8 assays after transfection with NC-plasmid, CNN3-plasmid, si-NC, si-CNN3#1 and si-CNN3#2 for 24 h. The OD value (450 nm) was detected every 24 h and continuously tested for 5 days. The results represent the mean ± SEM (n = 3). ***P < 0.001, ^###^P < 0.001. (**F**) Representative photographs of colony formation assays of SiHa and CaSki after transfection with NC-plasmid, CNN3-plasmid, si-NC, si-CNN3#1 and si-CNN3#2 are shown (upper panels), respectively. Quantification and statistics of colony area percentage per well is shown (lower graphs), mean ± SEM (n ≥ 3). *P < 0.05, **P < 0.01. (**G**) Boyden chamber migration and invasion assays were performed to detect the migration and invasiveness ability of SiHa and CaSki cells after transfection with NC-plasmid, CNN3-plasmid, si-NC, si-CNN3#1 and si-CNN3#2 for 24 h, respectively. Representative images (scale bars, 100 μm) and corresponding number (SiHa) or area (CaSki) of migrated (or invaded) cells from three independent experiments are shown, mean ± SEM (n ≥ 3), *P < 0.05, **P < 0.01, ***P < 0.001.

By analysis of an mRNA dataset named Pyeon Multi-cancer from the Oncomine database, we found that CNN3 mRNA expression was higher in 20 cervical cancer samples than in 8 normal cervix samples (Fig. 1A). Thus, we detected CNN3 mRNA expression in 7 pairs of cervical cancer and adjacent tissues, and found higher CNN3 mRNA expression in cervical cancer tissues than in adjacent tissues (Fig. 1B), consistent with the results from the publicly availabledataset. Then, we detected CNN3 protein in 10 cell lines, that included non-cancer cell lines (IMR-90, 293T, 293), cervical cancer cell lines (SiHa, Hela, CaSki), ovarian cancer cell lines (A2780, SKOV3, Caov3) and an osteosarcoma cell line (U2os). As Fig. 1C shows, CNN3 protein was highly expressed in the CaSki cell line (a cervical cancer cell line derived from a metastatic site), as well as in SKOV3, Caov3 and U2os cells. Considering that HPV oncoproteins act as drivers in the initiation and development of cervical cancer, we examined the effect of HPV16E5/E6/E7 on CNN3 expression in cervical cancer cell lines, SiHa and CaSki, and found decreased expression of CNN3 protein in both cells when they were transfected with E5 and E6/E7 siRNA, respectively (Fig. 1D). Furthermore, we observed that CNN3 was involved in the regulation of proliferation and metastasis in SiHa and CaSki cells. The CCK8 assays and colony formation assays showed that proliferation, migration and invasion were significantly enhanced when SiHa and CaSki cells were transfected with CNN3 plasmid, and were significantly suppressed when cells were transfected with two CNN3 siRNAs, compared to cells without gene transfection (Fig. 1E–G). Our results suggest that HPV16 oncoproteins may upregulateCNN3 expression and that CNN3 may play an oncogenic role in the development of cervical cancer.

### Stable CNN3 knockdown inhibits the growth and metastasis of xenografts of cervical cancer *in vivo*

**Figure 2:**
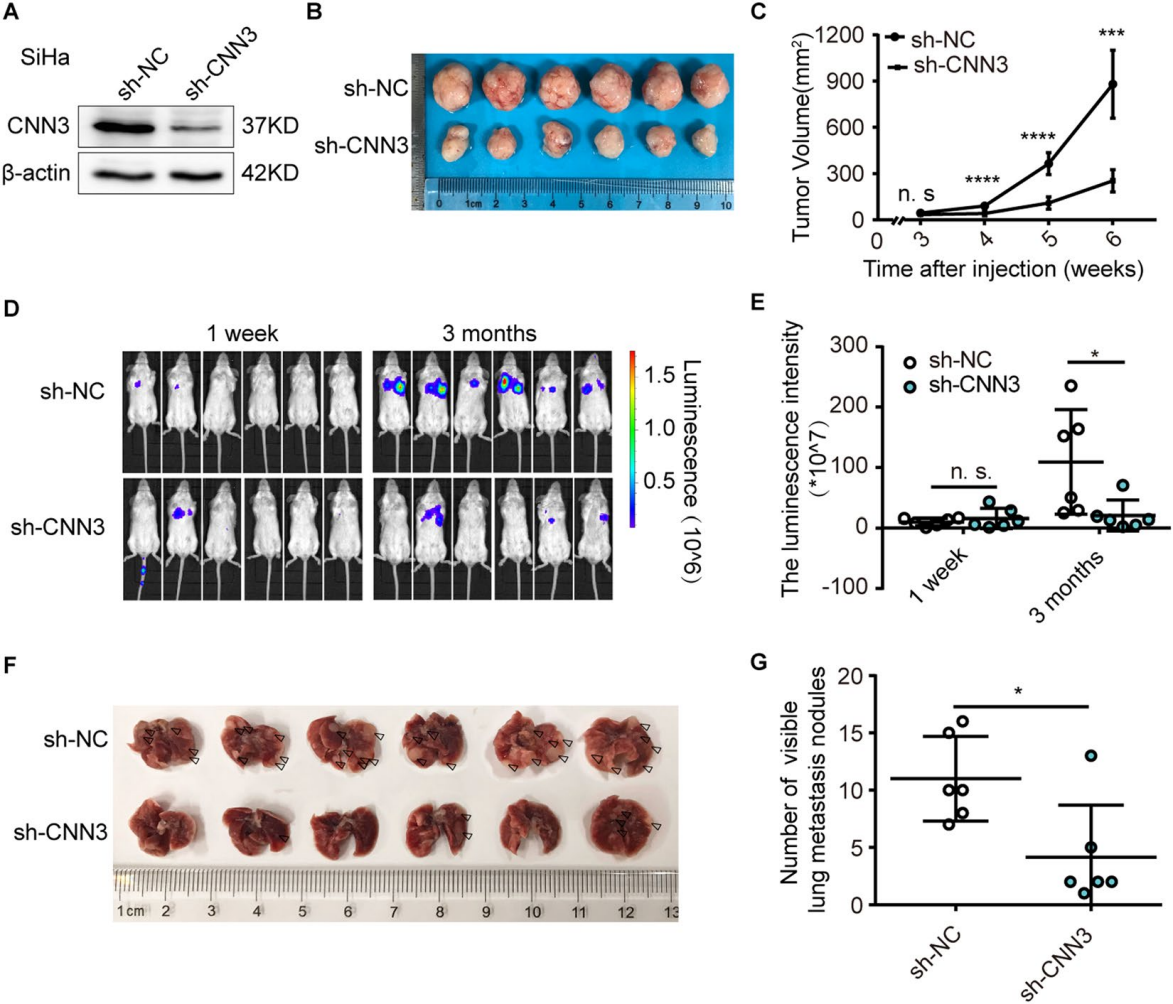
Stable CNN3 knockdown inhibits the growth and metastasis of xenografts of cervical cancer *in vivo*. (**A**) Immunoblot analysis of CNN3 protein in SiHa cells with sh-NC and sh-CNN3 lentivirus. The grouping of blots cropped from same parts of the same gel. Full-length blots are presented in Supplementary Fig. S4. (**B**,**C**) SiHa cells with sh-NC and sh-CNN3 were injected into nude mice (approximately 2.5 × 10^6^ cells, n = 6 in each group). Xenografts were extracted at week 7 when mice were sacrificed under anesthesia. The volume of subcutaneous tumours was tested weekly and calculated using the following formula: V = (L × W^2^)/2, mean ± SD. **P < 0.01, ***P < 0.001, ****P < 0.0001. (**D**,**E**) SiHa cells (approximately 2.5 × 10^6^ cells, n = 6 in each group) were intravenously injected into NOD/SCID mice via tail vein. The metastasis was weekly detected by measuring the bioluminescence with an IVIS Lumina LT system. The images and corresponding luminescence intensity at week 1 and week 12 after injection were displayed. Data represent mean ± SD (*P < 0.05). (**F**,**G**) Lungs were extracted at week 12 when mice were sacrificed under anesthesia. Lung metastases with white circular nodules (black arrow indicated) and corresponding numbers of metastatic nodules were showed. Data represent mean ± SD (*P < 0.05).

To confirm the oncogenic effect of CNN3 on the development of cervical cancer *in vivo*, we constructed a cell model (SiHa) with stable knockdown of CNN3 using sh-CNN3 lentivirus (Fig. 2A**)**. The same numbers of SiHa cells stably transfected with sh-CNN3 or sh-NC were subcutaneously injected into nude mice (n = 6 in each group). As shown in Fig. 2B,C, the sizes of xenografted tumours generated from cells with CNN3 knockdown were significantly smaller than those from cells without CNN3 knockdown. To confirm CNN3 promoting the metastasis of cervical cancer, we intravenously injected SiHa cells stably transfected with sh-CNN3 or sh-NC into scid mice via tail vein (n = 6 in each group), and found that the luminescence intensity was markedly decreased (Fig. 2D,E) and the numbers of visible lung metastases were significantly lower in the sh-CNN3#1 group at week 12 after transfection, compared to the controls (Fig. 2F,G), suggesting that CNN3 promotes the growth and metastasis of cervical cancer *in vivo*.

### RPLP1 is a candidate downstream gene in cervical cancer cells

**Figure 3:**
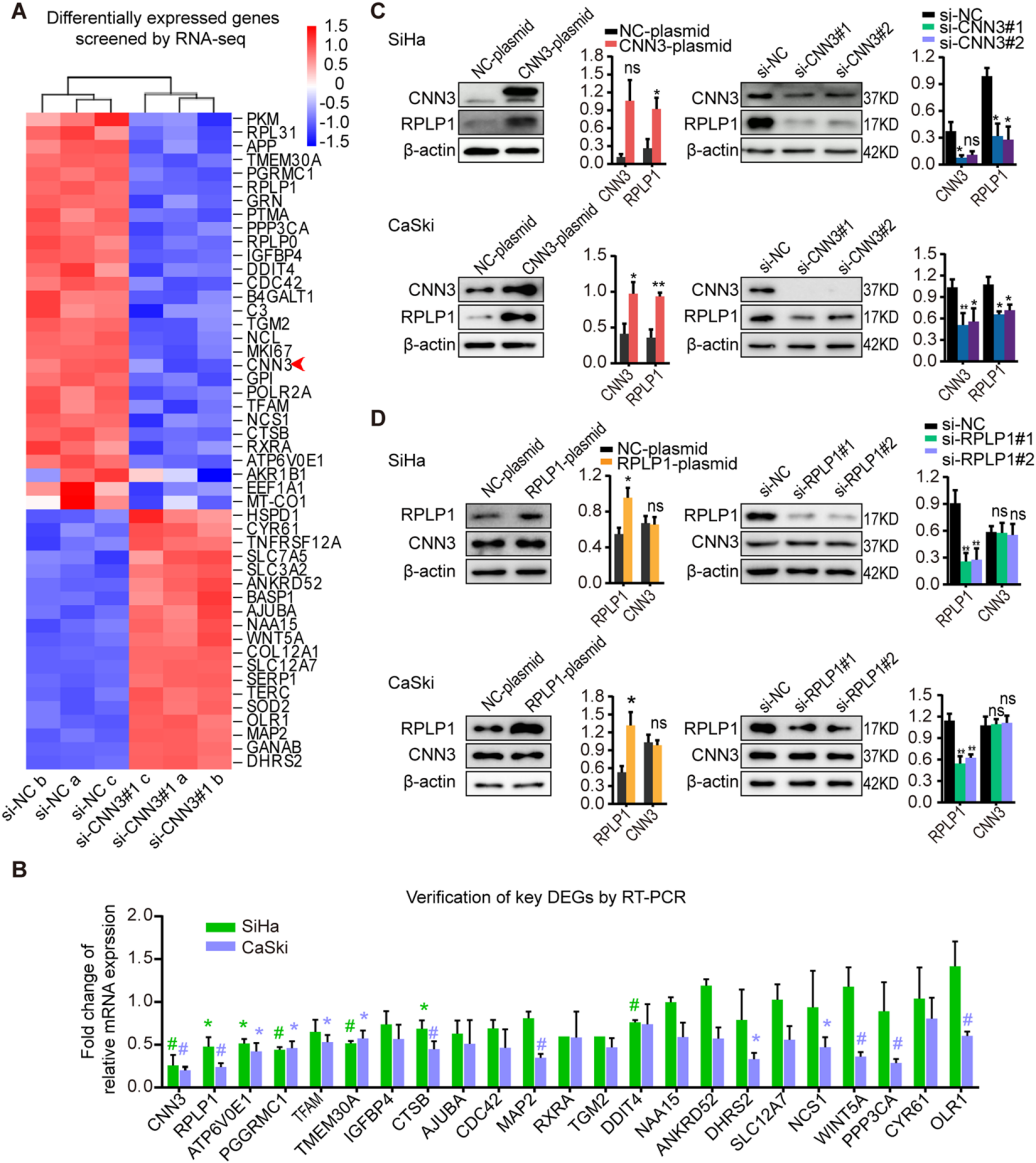
RPLP1 is a candidate downstream gene in cervical cancer cells. (**A**) RNA –seq data from the analysis between SiHa cells with si-NC and si-CNN3#1 at 48 h post-transfection. Shown in the cluster map are differentially expressed genes (P. adjusted ≤0.05) in common (n = 48). Of those, 19 were up-regulated and 28 down-regulated. Data shown are from three independent experiments. The arrow indicates CNN3. (**B**) After literature review, 23 key candidate downstream molecules were selected and validated by RT-PCR in SiHa and CaSki cells treated with si-NC and si-CNN3#1, respectively. Each column represents the fold change of relative CNN3 mRNA expression (i.e., relative CNN3 mRNA expression of si-CNN3#1 group/that of si-NC group). Data shown are mean ± SEM (n = 3), *P < 0.05, ^#^P < 0.01. (**C**) Immunoblot results of RPLP1 protein expression in cervical cancer cells with CNN3 upregulation and downregulation. At 48 h post-transfection, whole-cell lysates from SiHa and CaSki cells were collected to analyze the indicated proteins. β-actin was used as internal control. Quantification and statistical analysis of blots are shown (right graphs), mean ± SEM (n ≥ 3). *P < 0.05, **P < 0.01, ns indicates P > 0.05. Blots of CNN3 and β-actin cropped from same parts of the same gel, while blots of RPLP1 cropped from different parts of gel. The same is true for the blots in the following figures. Full-length blots are presented in Supplementary Fig. S5. (**D**) Representative immunoblot results of CNN3 protein expression from SiHa and CaSki cells with RPLP1 upregulation and downregulation. Quantification and statistical analysis of blots are shown (right graphs), mean ± SEM (n ≥ 3). *P < 0.05, **P < 0.01, ns indicates P > 0.05. Full-length blots are presented in Supplementary Fig. S6.

To explore the potential mechanism by which CNN3 affects the phenotypes of cervical cancer cells, we used RNA sequencing to detect changes in mRNA transcripts in SiHa cells with transfected si-NC or si-CNN3#1 (GEO Accession numbers: GSE134596). A total of 48 differentially expressed genes (DEGs) in common were found, with adjusted P values ≤ 0.05 in 3 biological replicates (Fig. 3A), which included 19 upregulated and 28 downregulated genes. Then, we conducted a literature review on these candidate downstream genes, and selected 23 key DEGs related to tumour proliferation, invasion and migration or both for validation. As shown in Fig. 3B, most of the DEGs presented consistent changes by RNA sequencing (except WINT5A and AJUBA), and among them, RPLP1 was the most significantly differentially expressed. RPLP1, an acidic ribosomal protein that recruits transcription factors in protein synthesis, has also been demonstrated to be related to primary cell transformation and breast cancer progression^16^. So we selected RPLP1 for further validation. We first examined the changes in RPLP1 protein expression in SiHa and CaSki cells with forced CNN3 expression, and found that the expression of RPLP1 protein was increased in both cells when CNN3 was overexpressed, and the opposite was observed in CNN3-silenced cells (Fig. 3C). In contrast, CNN3 protein expression remained unchanged when RPLP1 expression was modulated (Fig. 3D), suggesting that RPLP1 is a candidate downstream gene in cervical cancer cells.

### RPLP1 modulates the viability and activity of cervical cancer cells

**Figure 4:**
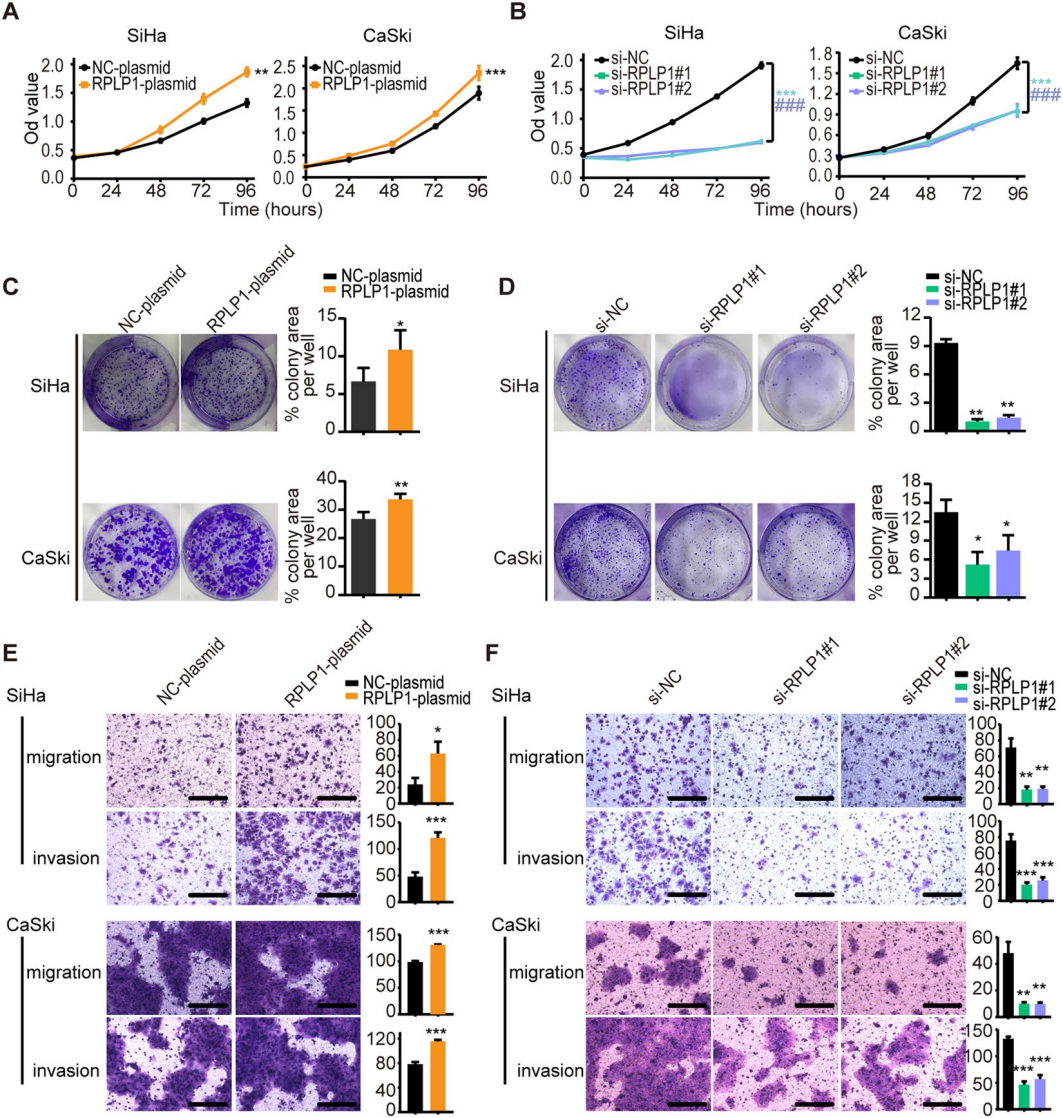
RPLP1 modulates the viability and activity of cervical cancer cells. (**A**,**B**) Cell proliferation of SiHa and CaSki cells were detected by CCK8 assays at 24, 48, 72, 96, 120 h after transfection with NC-plasmid, RPLP1-plasmid, si-NC, si-RPLP1#1, and si-RPLP1#2, respectively. Data represent mean ± SEM (n = 3). **P < 0.01, ***P < 0.001, ^###^P < 0.001. (**C**,**D**) Representative images of colony-formation assays for the proliferation in SiHa and CaSki cells with RPLP1 up-regulation and down-regulation are shown (left panels), respectively. Quantification and statistics of colony area percentage of each well are shown (right graphs), mean ± SEM (n ≥ 3). *P < 0.05, **P < 0.01. (**E**) Representative images of boyden chamber migration and invasion assays for the migration and invasion of SiHa and CaSki cells with NC-plasmid, RPLP1-plasmid (left panel), si-NC, si-RPLP1#1 and si-RPLP1#2 (right panel) for 24 h, respectively. Representative images (scale bars, 100 μm) and corresponding number (SiHa) or area (CaSki) of migrated (or invaded) cells from three independent experiments are shown, mean ± SEM, *P < 0.05, **P < 0.01, ***P < 0.001.

Using an RPLP1 expression plasmid and two different small interfering RNAs (si-RPLP1#1 and si-RPLP1#2) whose effects on CNN3 protein expression were confirmed in Fig. 3D, we investigated the role of RPLP1 in the viability and activity of SiHa and CaSki cells. CCK8, colony formation, and transwell assays showed that cellular proliferation, migration and invasion were enhanced in cervical cancer cells with RPLP1 overexpression (Fig. 4A,C,E), and, in contrast, were inhibited in cells with RPLP1 knockdown (Fig. 4B,D,F) compared to cells without any gene modulation. Our results suggest that RPLP1 plays the same role in cellular viability and activity as CNN3, and may participate in CNN3-regulated malignant behaviours in cervical cancer cells.

### RPLP1 participates in CNN3-modulated malignant behaviours in cervical cancer cells

**Figure 5:**
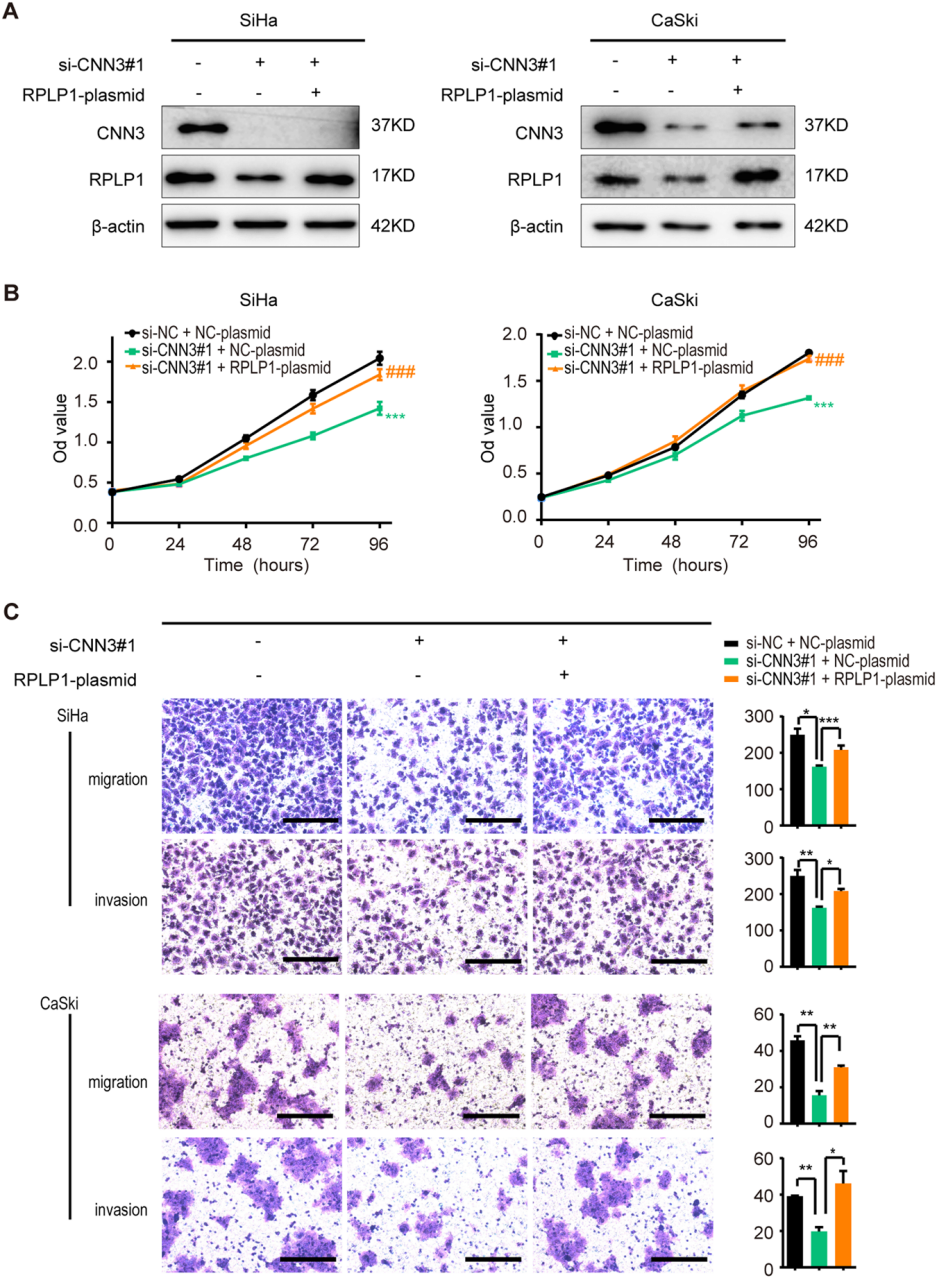
RPLP1 participates in CNN3-modulated malignant behaviours in cervical cancer cells. SiHa and CaSki cells were co-transfected with si-NC plus NC-plasmid, si-CNN3#1 plus NC-plasmid and si-CNN3#1 plus RPLP1-plasmid. (**A**) 48 h post-transfection, whole-cell lysates were obtained to analyze CNN3 and RPLP1 protein level with immunoblot assays. Representative results are shown. Full-length blots are presented in Supplementary Fig. S7. (**B**) CCK8 assays were applied to detect the proliferation of SiHa and CaSki cells. Data are shown of three independent experiments, mean ± SEM. ***P < 0.001, si-CNN3#1 plus NC-plasmid group *vs* si-NC plus NC-plasmid group; ^##^P < 0.01, ^###^P < 0.001, si-CNN3#1 plus RPLP1-plasmid group *vs* si-CNN3#1 plus NC-plasmid group. (**C**) Transwell migration and invasiveness assays were performed to detect cell migration and invasion. Left, representative images (scale bars, 100 um). Right, the corresponding histograms representative of three independent experiments (data are mean ± SEM, *P < 0.05, **P < 0.01, ***P < 0.001).

To confirm that CNN3 modulated malignant behaviours through the RPLP1 pathway, we conducted rescue experiments. SiHa and CaSki cells were co-transfected with si-NC plus NC-plasmid, si-CNN3#1 plus NC-plasmid, and si-CNN3#1 plus RPLP1-plasmid. The protein expression levels of CNN3 and RPLP1 proteins were detected to verify the success of the rescue experiments (Fig. 5A). As Fig. 5B,C showed, when we down-regulated CNN3 and upregulated RPLP1 expression simultaneously, the cellular proliferation, migration and invasion inhibited by CNN3 knockdown using si-CNN3#1 were partially or completely reversed by restoring RPLP1 expression. The results were similar when si-CNN3#2 was used (data shown in Supplementary Fig. S2).

## Discussion

The present study, to the best of our knowledge, is the first report to uncover the upregulated CNN3 expression in cervical cancer tissues, and its oncogenic role in promoting the proliferation, migration, and invasion of cervical cancer cells, and in facilitating the growth and lung metastasis of xenografted cervical cancer in immunodeficient mice.

To explore the mechanism by which CNN3 regulates the malignant behaviours of cervical cancer cells, we used the RNA-seq technique to identify potential downstream genes of CNN3. Furthermore, with “clusterprofiler”, a useful R package for gene set analysis and visualization, Gene Ontology (GO) analysis was performed of the DEGs and significantly enriched GO terms (p adjust < 0.05) were visualized in Supplementary Fig. S1. Of note, positive regulation of cell adhesion, monosaccharide metabolic process, regulation of developmental growth, neuron death, positive regulation of protein transport, and regulation of cell growth were identified as the main biological functions associated with DEGs. And these findings, to some extent, appeared to be consistent with malignant phenotypes induced by CNN3 overexpression.

Of those DEGs, further mRNA validation showed that RPLP1 was the most downregulated one in cells with CNN3 knockdown compared to cells without. Furthermore, we observed the effect of enforcedly changing RPLP1 expression on cellular proliferation, migration, and invasion in cervical cancer cells and found that the oncogenic function of RPLP1 is similar to that of CNN3. Rescue experiments further confirmed that RPLP1 restoration partially or completely reversed the proliferation, migration, and invasion inhibited by CNN3 knockdown in cervical cancer cells. Previous studies have verified that RPLP1, also known as ribosomal protein lateral stalk subunit P1, forms a lateral protuberance of the 60 S subunit of the ribosome, together with the other two isoforms RPLP0 and RPLP2, and interacts with the soluble translation factors, to regulate their activity during the course of protein synthesis^17^. However, substantial evidence has also shown that RPLP1 disruption does not result in abnormalities in overall mRNA translation or protein synthesis^18–22^, suggesting extra-ribosomal functions of RPLP1 in the cytoplasm. For instance, RPLP1 deletion in pMEFs (primary mouse embryonic fibroblasts) leads to proliferation arrest and premature senescence via dysregulation of key cell cycle and apoptosis regulators (cyclin A, cyclin E, p21CIP1, p27KIP1, p53), without alteration of global protein synthesis^21^. The absence of ribosomal P proteins, including RPLP1, results in reactive oxygen species (ROS) accumulation and MAPK1/ERK2 signalling pathway activation, thereby leading to autophagy^18^. In addition, RPLP1 mediates cell invasion by affecting the epithelial-mesenchymal transition in triple-negative breast cancer cells^16^. Thus, our results, combined with those from previous studies, suggest that RPLP1, as a downstream gene of CNN3, plays a key role in regulating malignant behaviours of cervical cancer cells.

As noted above, CNN3 affected RPLP1 mRNA expression in cervical cancer cells. To explore the potential mechanism of CNN3-mediated regulation of RPLP1 expression, we examined the distribution of CNN3 in wild-type cervical cancer cells and found a trace amount of CNN3 proteins in the nucleus, which was in line with the immunofluorescence results in other studies^12,23,24^.

In conclusion, CNN3 acts as an oncogene by promoting invasion and migration in cervical cancer cells and accelerating the growth and metastasis of xenografted cervical cancer in mouse model. RPLP1 functions as CNN3 does, and participates in CNN3 promoting malignant behaviours by affecting RPLP1 mRNA expression. Our findings suggest that CNN3 may serve as a potential therapeutic target for advanced stage cervical cancer.

## Materials and Methods

### Cell lines and cell culture

The human SiHa, CaSki cell lines were purchased from ATCC. SiHa cells were cultured in DMEM Medium while CaSki maintained in RPMI 1640 medium supplemented with 10% fetal bovine serum and incubated in a 37 °C humidified incubator with 5% CO_2_.

### Transient transfection

DNA constructs were transiently transfected into SiHa and CaSki cells using X-tremeGENE HP DNA Transfection Reagent (Roche, Basel, Switzerland). In brief, appropriate amount of cells were seeded in 6-well plates. Make sure cells are at 70–80% confluence when transfected (at log-growth phase). Cells of each well were transfected with 2.5 ug of DNA according to the manufacturer’s instruction. The ratio of transfection regent: DNA is 2: 1 for SiHa and 3: 1 for CaSki. 24 h after transfection, the medium was replaced by fresh complete medium.

For siRNA transfection, we used DharmaFECT™ Transfection Reagents (Thermo, Waltham, MA, USA). According to the instructions, when the cells proliferated to ~60% confluence, 10 ul of 20 uM siRNA was added to 190 ul of OPTI-MEM to prepare solution A, and 6 ul of the transfection reagent was added to 194 ul of OPTI-MEM to prepare solution B. Mix gently and incubate at room temperature for 5 min. Then, the solution A was added to the solution B and mixed. After incubating at room temperature for 15 min, 200 ul of the transfection mixture was added to each well. 20 h after transfection, the medium containing transfection reagents was replaced by fresh medium. SiRNAs specific for CNN3 and RPLP1 were purchased from GenePharma (Shanghai, China) and RiboBio (Guangzhou, China) and were described as follows:

si-CNN3#1 sense: GCAGAUGGGAACCAACAAATT;

si-CNN3#2 sense: GTCAACGAGTCCTCACTGA;

si-RPLP1#1 sense: GGAAGCAAAGAAAGAAGAA;

si-RPLP1#2 sense: GAAAGTGGAAGCAAAGAAA;

si-NC sense: UUCUCCGAACGUGUCACGUTT

si-E5 sense: TGGTATTACTATTGTGGATAA

si-E6/E7 sense: GGUUGUGCGUACAAAGCAC

For RNA interference sequencing targeting HPV oncoproteins 16E5, E6/E7, are derived from previous studies^25,26^.

### RNA extraction and quantitative RT-PCR

Total RNA was extracted from SiHa and CaSki cells using Trizol reagent (Invitrogen, New York, USA) according to the manufacturer’s instructions, and then the reverse transcription reaction was synthesized using the PrimeScript^TM^ RT reagent Kit with gDNA Eraser (Takara, Dalian, China). The sequences of all the primers amplified for qRT-PCR were listed after:

β-actin forward 5′-AACTCCATCATGAAGTGTGACG-3′

reverse 5′-GATCCACATCTGCTGGAAGG-3′

RPLP1 forward 5′-AGCCTCATCTGCAATGTAGGG-3′

reverse 5′-TCAGACTCCTCGGATTCTTCTTT-3′

CNN3 forward 5′-GCAGGGATGTTAGCACCAGGTA-3′

reverse 5′-CTGTTCCTTGGCTTCCGTTGTG-3′

Real-time PCR was performed using TB Green™ Premix Ex Taq™ (TaKaRa, Dalian, China), by a ABI-7500 qRT-PCR system thermal cycler. Human β-actin relative mRNA level was applied as an internal control, and the relative quantity was calculated using the 2^−ΔΔCT^ method.

### Western blotting

SiHa and CaSki cells were cultured in 6-well plates for about 48 h after transfection and were harvested and lysed in 200 ul RIPA lysis buffer (strong, without inhibitors, Beyotime, Shanghai, China) with 1% protease inhibitors Cocktail and 1% phosphatase Inhibitor Cocktail (EDTA-Free, 100X in DMSO, Bimake, USA) for 30 min. After being centrifuged, the concentrations of total protein in cell lysates were determined by bicinchonininc acid assays. The cell lysates (20 μg/lane) were separated by 12% SDSpolyacrylamide gel electrophoresis and transferred onto PVDF membranes (0.22 um). The membranes were blocked with skim dry milk (5%) in Tris-buffered saline containing 0.05% Tween-20 and incubated with primary antibodies at 4 °C overnight. After being washed, the bound antibodies were detected with HRP-conjugated second antibodies. Finally, the blots were visualized using the enhanced chemiluminescence (ECL) method. The primary antibodies used including anti-CNN3 (1:1000, ab15142, Abcam, Cambridge, MA, USA), anti-RPLP1 (1:1000, MA5-24411, ThermoFisher, Waltham, MA, USA), anti-β-actin (1:5000, 70-ab008-100, MULTISCIENCES, Hangzhou, China), anti-P53 (1:1000, sc-126, SANTA CRUZ, California, USA).

### Cell proliferation and colony formation assay

CCK8 analysis and colony formation assays were carried out to determine the function of CNN3 and RPLP1 on cell proliferation. 24 h after transfection, for CCK8 analysis, approximately 3.5 × 10^3^ cells were seeded in each well of 96-well plates and CCK8 solution (Dojindo Laboratories, Kumamoto, Japan) was added 0, 24, 48, 72, and 96 h after seeding. After that, cells were incubated at 37 °C for 2 h. The absorbance at 450 nm was measured. For colony formation assays, 1 × 10^3^ cells were cultured in 6-well plates at 37 °C for 10 days, and then washed twice with PBS, fixed, and stained with 1% crystal violet in 25% methanol. And the percentage of cell colony area of each well was assessed by Image J.

### Cell migration and invasion assay

Cell migration and invasion assays were carried out using 24-well transwell plates (8 μm pore, Corning Costar, New York, USA) with a pore size of 8 μm. The transwell filter inserts were coated with (invasion) or without (migration) Matrigel (BD Biosciences, 1:8 diluted). Cells were transfected with NC-plasmid or CNN3-plasmid, respectively. About 24 h later, 2 × 10^5^ cells were seeded in the upper chambers with OPTI-MEM. The lower chambers were filled with medium supplemented with 10% FBS. After incubated at 37 °C with 5% CO_2_ for 5 and 8 h (SiHa) or 12 and 24 h (CaSki), respectively, the cells that had traversed the membrane were fixed in 90% ethanol and stained with 0.5% crystal violet solution while the cells in the upper chamber were carefully removed using a cotton swab, followed by photo-imaging. Finally, we calculated the average number of SiHa cells in 5 fields, while calculated the transmembrane area of CaSki cells in 5 fields.

### RNA-seq analysis

SiHa cells transfected with si-NC or si-CNN3#1 were cultured for 48 h after transfection. Samples of three independent assays from each group were prepared. Total RNA was prepared using Trizol method according to the manufacturer’s instructions. RNA quality was evaluated using a RNA NanoPhotometer^®^ spectrophotometer (IMPLEN, Munich, Germany). And high-quality RNA from si-NC and si-CNN3#1 groups was used for transcriptome sequencing. The libraries were sequenced on an Illumina HiSeq/MiSeq platform using a sequencing by synthesis strategy. The original data were then purified by error rate check, GC distribution check and raw data screening for subsequent analysis. And at least 7.25G clean data were obtained from each sample. Then, clean reads were aligned to the reference genome using Topha2 algorithm. Reads count of mapped genes were counted and FPKM were introduced to evaluate the gene expression level, and differential expression analysis of two conditions was performed using the DESeq package. Finally, genes with an adjusted P-value < 0.05, were considered to be differentially expressed.

### Stable cell line generation

To obtain luciferase stably expressing SiHa cells for tumour formation and metastasis model construction, we infected luciferase-stably-expressing SiHa cells with a lentiviral construct (hU6-MCS-Ubiquitin-Luc_firefly-IRES-puromycin) expression sh-CNN3 targeting 5′-GCAGATGGGAACCAACAAA-3′ or a scrambled shRNA (control). The lentiviruses were purchased from Genechem (Shanghai, China). And the MOI for infection is 25. 24 h post infection, cells were selected with 0.9 ug/ml puromycin, with medium refreshed every 2 days. 5 days later, the concentration of puromycin was halved and maintained for 2 weeks. Finally, when the cells grown to the desired number, they were digested, washed with PBS, and resuspended in PBS to a final density of 2.5 × 10^7^ cells/ml.

### Construction of tumour-formation and metastatic mice models

All animal experimental protocols were performed in accordance with Animal Research Reporting *In Vivo* Experiments (ARRIVE) guidelines and were approved by Animal Ethical and Welfare Committee of Zhejiang Chinese Medical University (Approval No. IACUC-20180807-05). For tumour formation assays, we randomly divided 12 female BALB/c nude mice (Silaike Experiment Animal Co., Ltd., Shanghai, China) of 3–4 weeks into 2 groups, and 100 ul cell suspension of SiHa stably transfected with sh-NC and sh-CNN3 were subcutaneous injected into the skin of right upper limb respectively. Tumor growth were measured weekly and the volumes of the xenograft tumours were calculated using the following formula: length × width × width × 0.5. And 7 weeks later, the mice were sacrificed under CO_2_ anesthesia and the subcutaneous tumours were obtained.

For construction of metastatic mice models, we randomly divided 12 female CB-17 Scid mice (Silaike Experiment Animal Co., Ltd., Shanghai, China) of 3–4 weeks into 2 groups and same amount of stable transfected SiHa cells were injected intravenously respectively. In both two group, the mice were administered 150 mg/kg D-luciferin (Yeasen, Shanghai, China) by intraperitoneal injection and performed *in vivo* imaging 10 min later weekly with application of IVIS Lumina LT (PERKINELMER, Waltham, Massachusetts, USA). And the ROI of mice were analyzed using LIVINGIMAGE software.

### Statistical analysis

All statistics were conducted with SPSS version 20.0 (IBM Corp, USA). In addition to the cell viability evaluated by One-way repeated measurement analysis of variance, all the other data in this paper were estimated using the Student’s t-test.

## Supplementary information

Supplementary Information.

## Data Availability

The datasets generated during and/or analyzed during the current study are available from the corresponding author on reasonable request.
