# Supplementary material for: CNN3 acts as a potential oncogene in cervical cancer by affecting RPLP1 mRNA expression
Source: Sci Rep. 2020 Feb 12;10:2427. doi: 10.1038/s41598-020-58947-y (PMC7016181; doi:10.1038/s41598-020-58947-y)
Supplement: Supplementary file 1 — Supplementary Information. [file 41598_2020_58947_MOESM1_ESM.pdf]

Supplementary Information for

CNN3 acts as a potential oncogene in cervical cancer by affecting RPLP1 mRNA expression

Lili Xia[1*], Yongfang Yue[1*], Mingyue Li[1], Ya-Nan Zhang[1], Lu Zhao[1], Weiguo Lu[2*], Xinyu Wang[2*], and Xing Xie[2*]

[1] Women's Reproductive Health Laboratory of Zhejiang Province, Women's Hospital, School of Medicine, Zhejiang University, Hangzhou 310006 Zhejiang, China.
[2] Department of Gynecologic Oncology, Women's Hospital, School of Medicine, Zhejiang University, Hangzhou 310006 Zhejiang, China.

Corespondence author: Xing Xie
Corresponding email: xiex@zju.edu.cn

SUPPLEMENTAI FIGURE LEGENDS

Supplementary Figure S1

Gene Ontology analysis of DEGs screened by RNA-seq

GO enrichment analysis was performed using “clusterProfiler”, which is a very useful R package for gene set analysis and visualisation. Significantly enriched GO terms (p. adjust ＜ 0.05） which represents the main biological functions of DEGs were visualized. Detail information of GO results is provided in Supplementary Table. **S1.**

Supplementary Figure S2

RPLP1 participates in CNN3-modulated malignant behaviours in cervical cancer cells.

SiHa and CaSki cells were co-transfected with si-NC plus NC-plasmid, si-CNN3#2 plus NC-plasmid, and si-CNN3#2 plus RPLP1-plasmid. (**A**) 48 h post-transfection, whole-cell lysates were obtained to analyze CNN3 and RPLP1 protein levels with immunoblot assay. Representative results are shown. (**B**) CCK8 assay were applied to detect the proliferation of SiHa and CaSki cells. Data are shown of three independent experiments, mean ± SEM. ***P < 0.001, si-CNN3#2 plus NC-plasmid group *vs* si-NC plus NC-plasmid group; $^{\#\#}$P < 0.01, $^{\#\#\#}$P < 0.001, si-CNN3#2 plus RPLP1-plasmid group *vs* si-CNN3#2 plus NC-plasmid group. (**C)** Transwell migration and invasiveness assays were performed to detect cell migration and invasion. Left, representative images (scale bars, 100 um). Right, the corresponding histograms representative of three independent experiments (mean ± SEM, *P < 0.05, **P < 0.01, ***P < 0.001).

Supplementary Figure S3

Full immunoblot exposures for results shown in Figure 1C and 1D.

Lanes highlighted by a star represent samples related to this study.

Supplementary Figure S4

Full immunoblot exposures for results shown in Figure 2A.

Lanes highlighted by a star represent samples related to this study.

Supplementary Figure S5

Full immunoblot exposures for results shown in Figure 3C.

Lanes highlighted by a star represent samples related to this study.

Supplementary Figure S6

Full immunoblot exposures for results shown in Figure 3D.

Lanes highlighted by a star represent samples related to this study.

Supplementary Figure S7

Full immunoblot exposures for results shown in Figure 5A.

Lanes highlighted by a star represent samples related to this study.

Supplementary Figure S8

Full immunoblot exposures for results shown in Supplementary Figure S2.

Lanes highlighted by a star represent samples related to this study.

Supplementary Figure S1

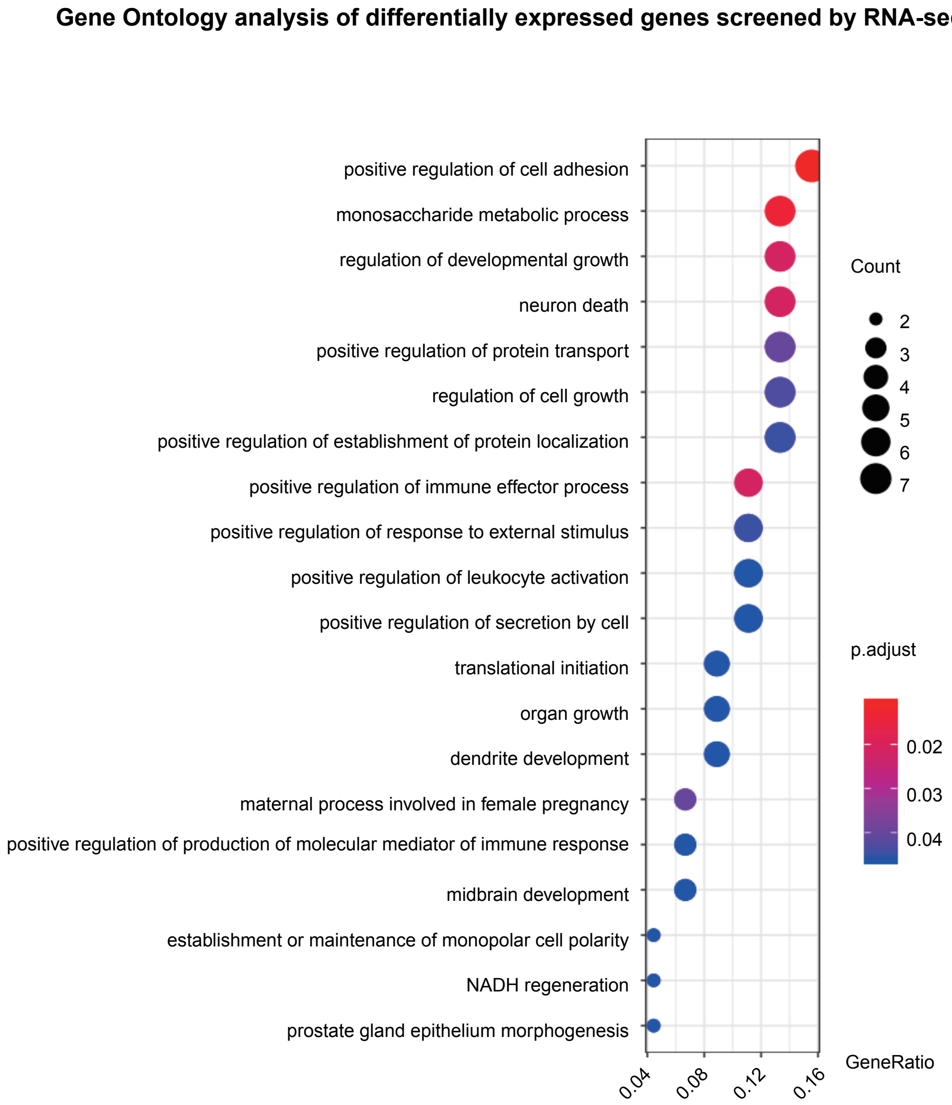

Supplementary Figure S2

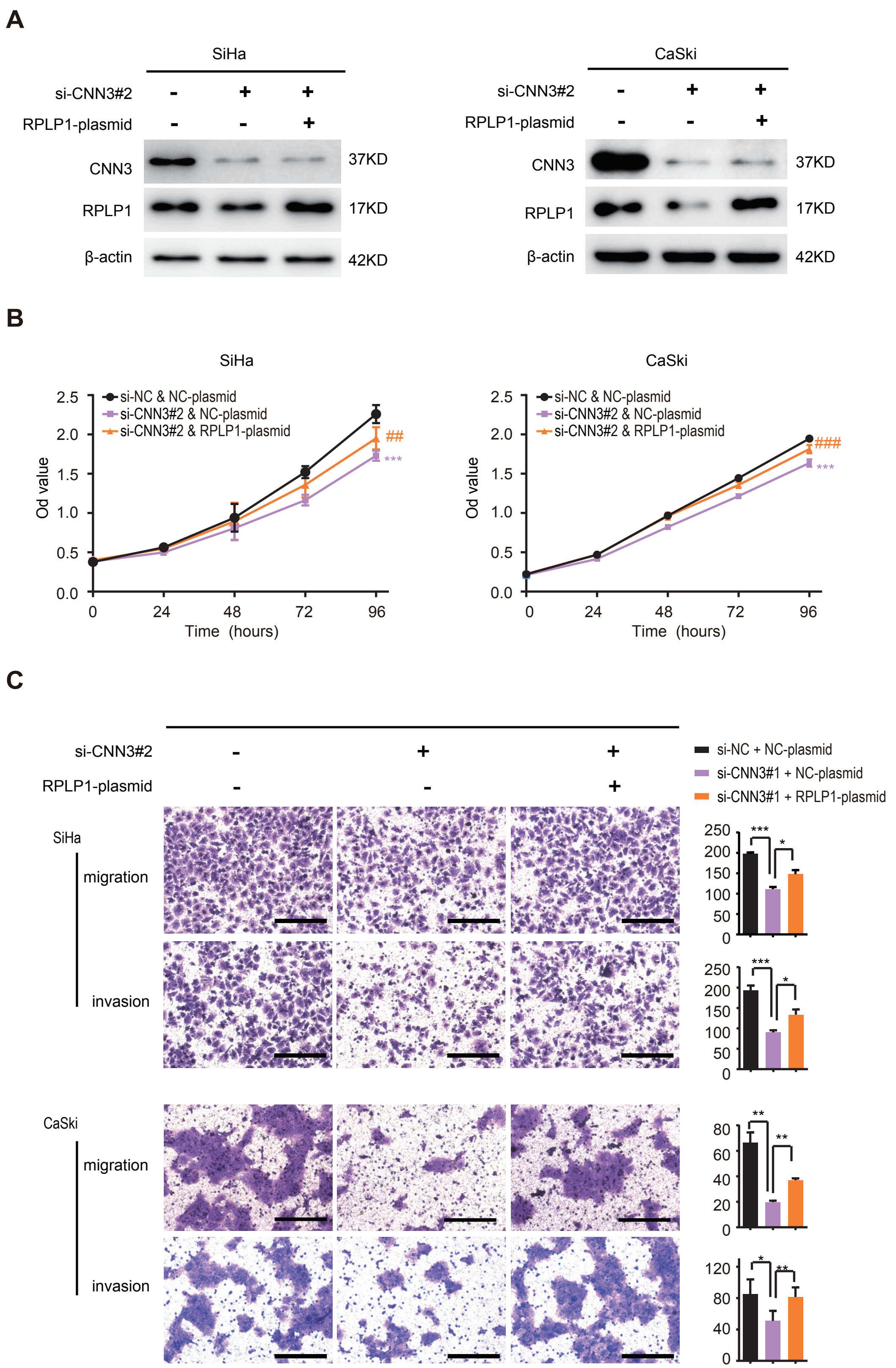

Supplementary Figure S3

Fig. 1C

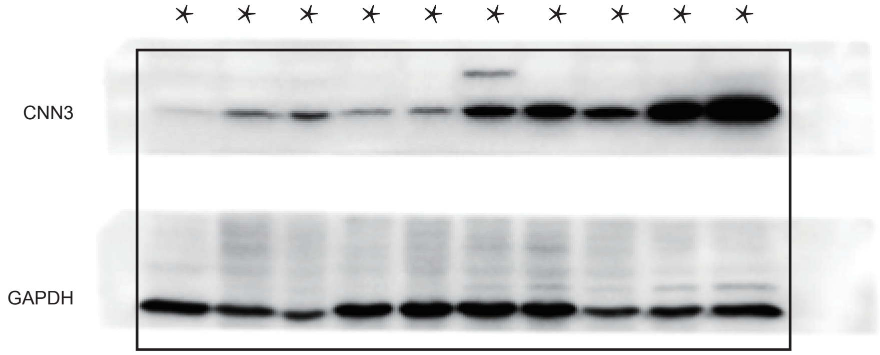

Fig. 1D（left panel）

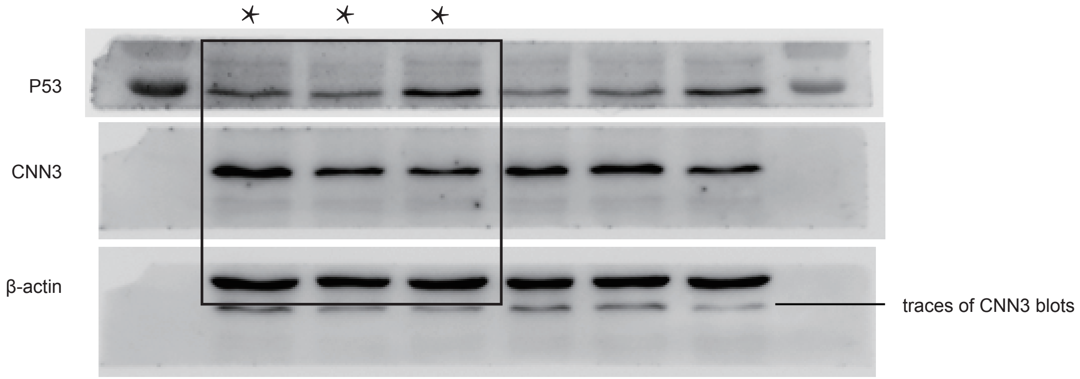

Fig. 1D（right panel）

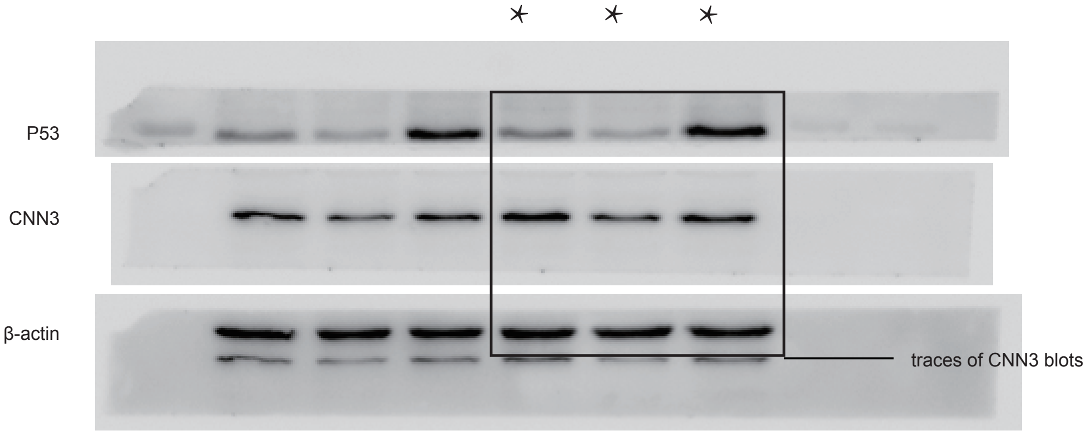

Supplementary Figure S4

Fig. 2A

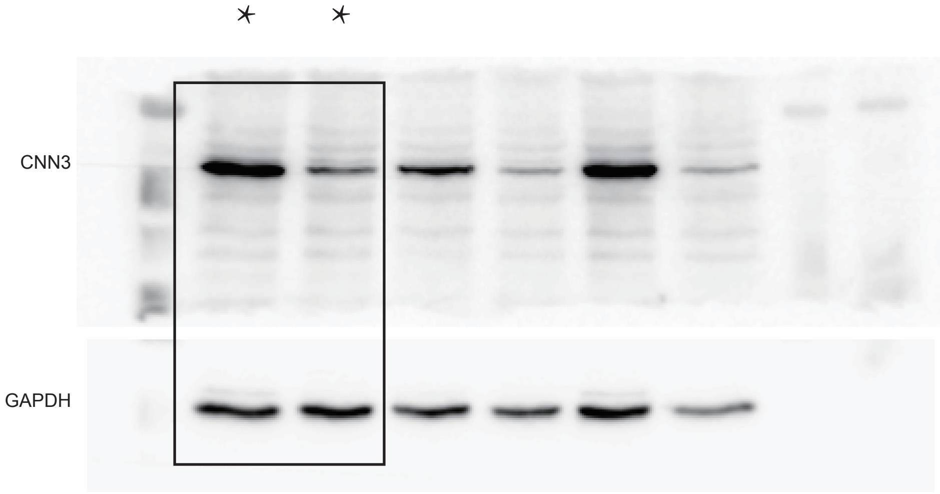

Supplementary Figure S5

Fig. 3C (upper panel)

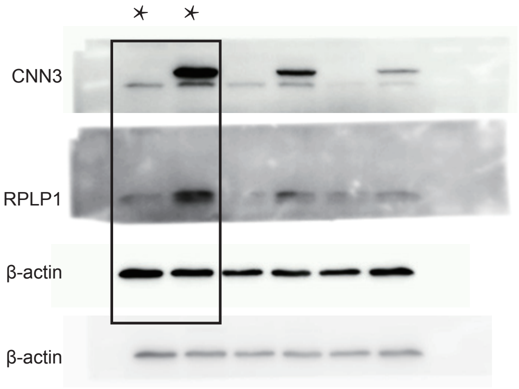

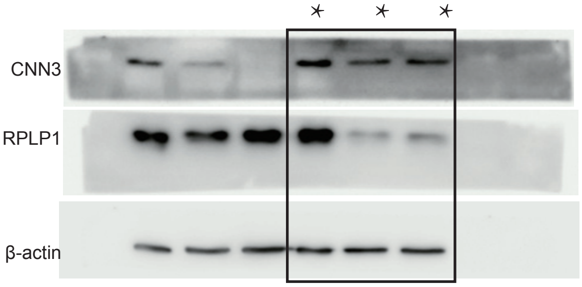

Fig. 3C (lower panel)

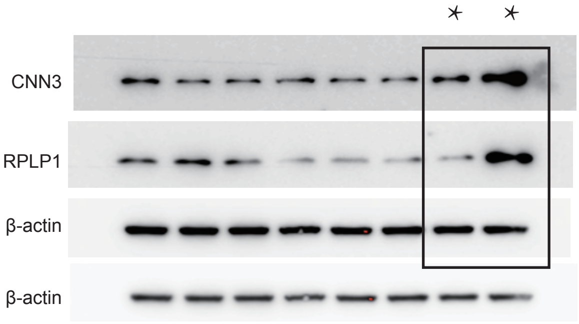

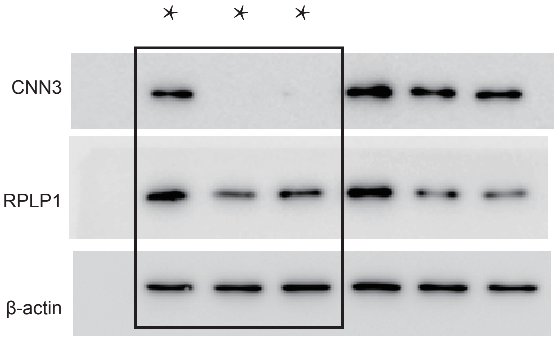

Supplementary Figure S6

Fig. 3D (upper panel)

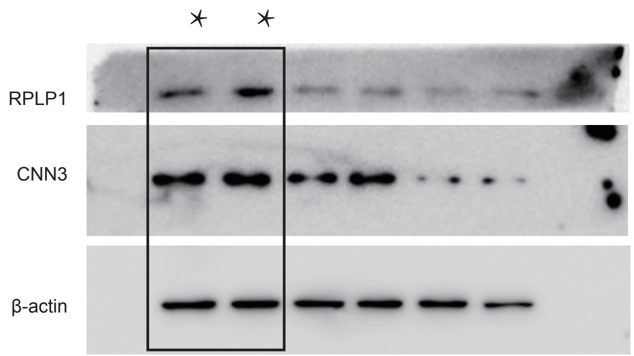

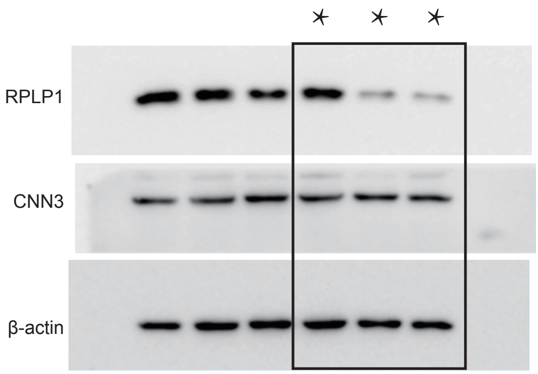

Fig. 3D (lower panel)

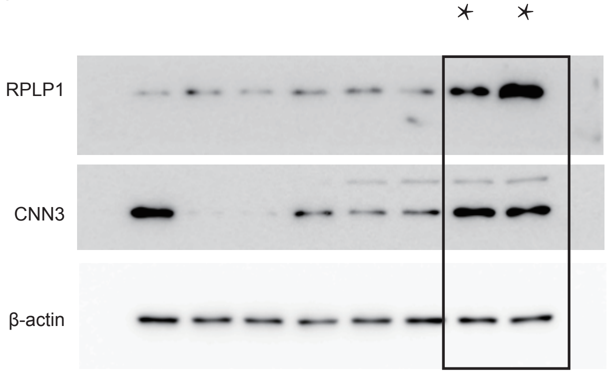

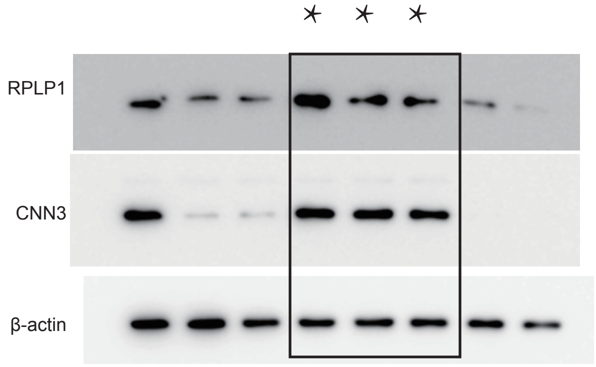

Supplementary Figure S7

Fig. 5A（left panel）

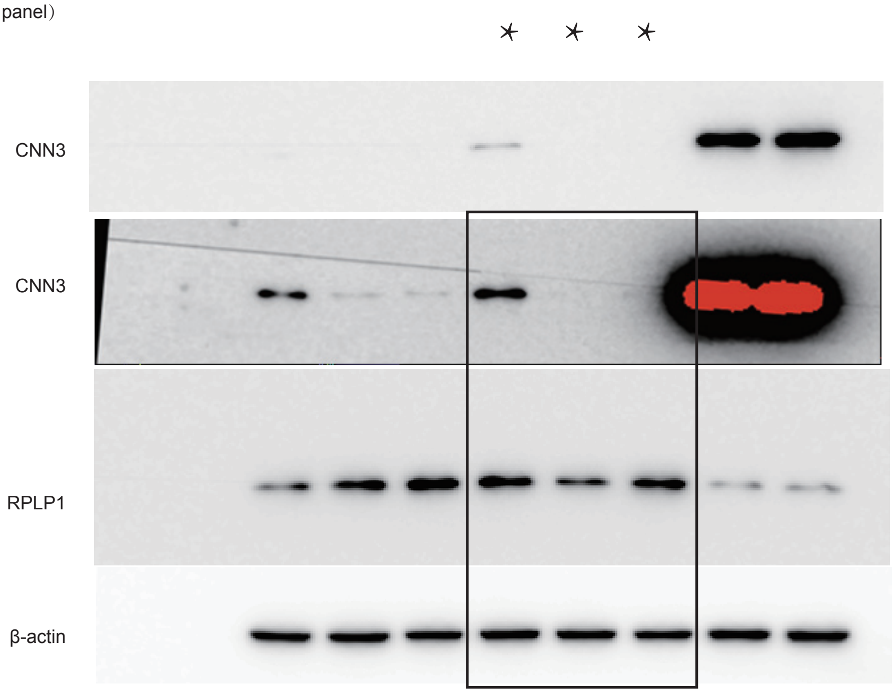

Fig. 5A（right panel）

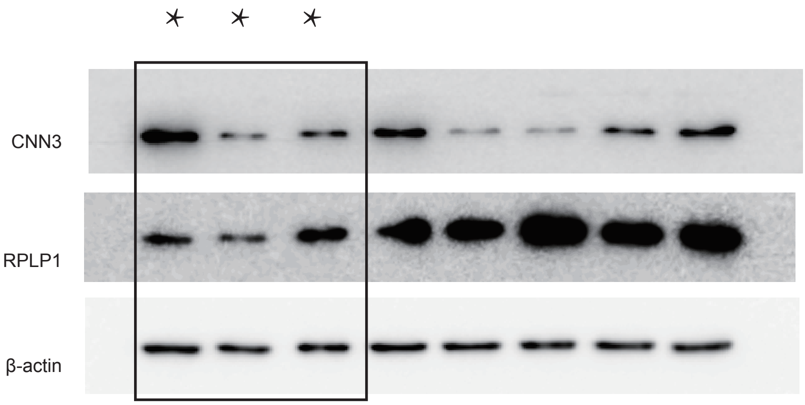

Supplementary Figure S8

Fig. S2A （left panel）

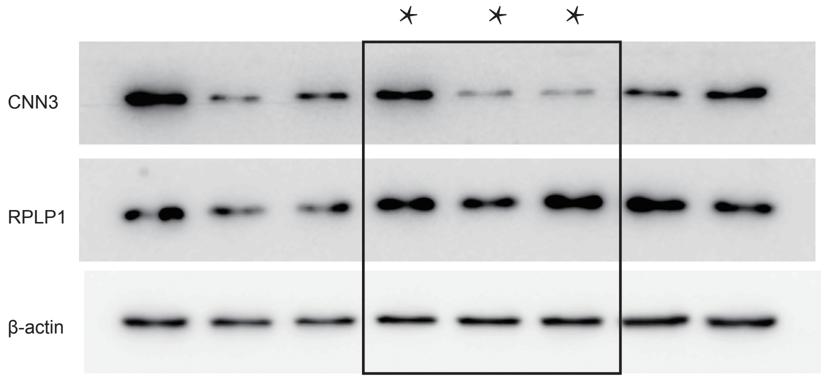

Fig. S2A （right panel）

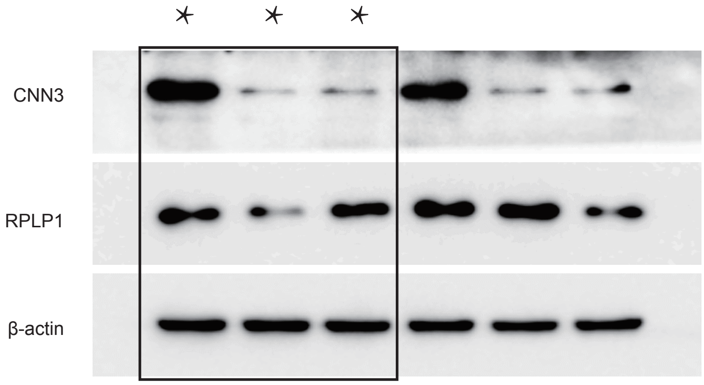

Supplementary Table S1 Significantly enriched GO terms of DEGs screened by RNA-seq

Description	GeneRatio	p.adjust	geneID	Count
positive regulation of cell adhesion	7/45	0.010699895	TFRC/CYR61/CDC42/HSPD1/TGM2/PPP3CA/WNT5A	7
monosaccharide metabolic process	6/45	0.013804132	IGFBP4/SERP1/PKM/GPI/AKR1B1/B4GALT1	6
positive regulation of immune effector process	5/45	0.021375155	TFRC/GPI/HSPD1/C3/WNT5A	5
regulation of developmental growth	6/45	0.021375155	SERP1/TNFRSF12A/APP/MAP2/BASP1/WNT5A	6
neuron death	6/45	0.021375155	DDIT/APP/GPI/CDC42/HSPD1/WNT5A	6
positive regulation of protein transport	6/45	0.039996592	SERP1/GPI/TMEM30A/CDC42/HSPD1/WNT5A	6
maternal process involved in female pregnancy	3/45	0.039996592	AKR1B1/CTSB/RXRA	3
regulation of cell growth	6/45	0.043015362	IGFBP4/TNFRSF12A/CYR61/CDC42/MAP2/WNT5A	6
positive regulation of establishment of protein localization	6/45	0.044904876	SERP1/GPI/TMEM30A/CDC42/HSPD1/WNT5A	6
positive regulation of response to external stimulus	5/45	0.044904876	APP/HSPD1/TGM2/C3/WNT5A	5
establishment or maintenance of monopolar cell polarity	2/45	0.046085063	CDC42/WNT5A	2
translational initiation	4/45	0.046085063	RPL31/RPLP0/RXRA/RPLP1	4
organ growth	4/45	0.046085063	SERP1/COL12A1/BASP1/RXRA	4
positive regulation of production of molecular mediator of immune response	3/45	0.046085063	TFRC/GPI/WNT5A	3
midbrain development	3/45	0.046085063	CDC42/BASP1/WNT5A	3
NADH regeneration	2/45	0.046085063	PKM/GPI	2
prostate gland epithelium morphogenesis	2/45	0.046085063	RXRA/WNT5A	2
dendrite development	4/45	0.046085063	APP/CDC42/MAP2/PPP3CA	4
positive regulation of leukocyte activation	5/45	0.046085063	TFRC/APP/CDC42/HSPD1/WNT5A	5
positive regulation of secretion by cell	5/45	0.046085063	SERP1/NCS1/GPI/HSPD1/WNT5A	5
prostate gland morphogenesis	2/45	0.046085063	RXRA/WNT5A	2
developmental cell growth	4/45	0.046085063	TNFRSF12A/APP/MAP2/WNT5A	4
developmental growth involved in morphogenesis	4/45	0.046085063	TNFRSF12A/APP/MAP2/WNT5A	4
positive regulation of cell activation	5/45	0.046085063	TFRC/APP/CDC42/HSPD1/WNT5A	5
maternal placenta development	2/45	0.047348942	CTSB/RXRA	2
positive regulation of cartilage development	2/45	0.047686207	CYR61/WNT5A	2
regulation of gene silencing	3/45	0.047686207	AJUBA/POLR2A/PPP3CA	3
positive regulation of secretion	5/45	0.047688347	SERP1/NCS1/GPI/HSPD1/WNT5A	5
formation of primary germ layer	3/45	0.048120242	GPI/COL12A1/WNT5A	3
positive regulation of cell-cell adhesion	4/45	0.049218926	TFRC/CDC42/HSPD1/WNT5A	4
positive regulation of defense response	5/45	0.049385907	APP/HSPD1/TGM2/C3/WNT5A	5
reproductive structure development	5/45	0.049385907	CYR61/CTSB/BASP1/RXRA/WNT5A	5
establishment of cell polarity	3/45	0.049385907	CDC42/MAP2/WNT5A	3
cardiac chamber morphogenesis	3/45	0.049385907	CYR61/RXRA/WNT5A	3
positive regulation of growth	4/45	0.049729079	SERP1/TNFRSF12A/CDC42/BASP1	4
regulation of immune effector process	5/45	0.049800665	TFRC/GPI/HSPD1/C3/WNT5A	5
regulation of production of molecular mediator of immune response	3/45	0.049800665	TFRC/GPI/WNT5A	3
